# Floral scents, cozy shelter, and edible spathes: brood-site weevil pollination in *Stenospermation weberbaueri* (Araceae)

**DOI:** 10.1186/s40529-025-00468-w

**Published:** 2025-10-10

**Authors:** Luna Ibáñez, Eliana Álvarez-Valdez, Artur Campos Dália Maia, Luis Alberto Núñez-Avellaneda, Gonzalo Taborda-Ocampo, Alejandro Zuluaga-Trochez, Natalia Castaño-Rubiano

**Affiliations:** 1 Semillero de Investigación en Plantas y Afines PHYTOS, Grupo de Investigación en Biodiversidad y Recursos Naturales BIONAT, Departamento de Ciencias Biológicas, Facultad de Ciencias Exactas y Naturales, Universidad de Caldas, 170004 Manizales, Colombia; 2 Departamento de Ciencias Biológicas, Facultad de Ciencias Exactas y Naturales, Herbario de la Universidad de Caldas - FAUC, Grupo de Investigación Biodiversidad y Recursos Fitogenéticos - BIONAT, Universidad de Caldas, 170004 Manizales, Colombia; 3 Facultad de Ciencias, Programa de Maestría en Ciencias Biológicas, departamento de Ciencias Biológicas Universidad de Los Andes, Bogotá, Colombia; 4 Laboratorio de Ecología de Bosques Tropicales y Primatología (LEBTYP), Universidad de Los Andes, Bogotá, Colombia; 5 Laboratorio de Investigación en Química Analitica, Grupo de Investigación de Cromatografía y Técnicas Afines - GICTA, Departamento de Química, Facultad de Ciencias Exactas y Naturales, Universidad de Caldas, 170004 Manizales, Colombia; 6 Programa de Pós-Graduação Em Biologia Animal, Centro de Biociências, Departamento de Zoologia, Universidade Federal de Pernambuco, Recife, 50670901 Brazil; 7 Programa de Pós-Graduação Em Biologia Vegetal, Centro de Biociências, Departamento de Botânica, Universidade Federal de Pernambuco, Recife, 50670901 Brazil; 8 Grupo de Investigación Ecotonos, Programa de Biología, Universidad de Los Llanos, Villavicencio, Colombia; 9 Departamento de Biología, Universidad del Valle, 76001 Cali, Colombia; 10 Herbario Luis Sigifredo Espinal Tascón, Universidad del Valle, 76001 Cali, Colombia

**Keywords:** Cantharophily, Floral visitors, Pollination ecology, Semiochemicals, Volatile organic compounds, Dynamic headspace, Thermogenesis-marked landing sites

## Abstract

**Background and Objectives:**

Araceae family is known for its entomophilous reproductive strategies, involving olfactory-driven pollinator attraction and diverse floral rewards. In the Neotropics, genera with unisexually-flowered inflorescences, feature short anthesis and intense thermogenesis, while those with bisexual flowers typically have longer anthesis and varied strategies. However, Monsteroideae, with short anthesis, remains an exception and is underexplored. This study focuses on the floral morphology, pollination ecology, and reproductive strategies of *Stenospermation weberbaueri* to enhance understanding of Neotropical aroid dynamics.

**Material and methods:**

A natural population of *S. weberbaueri* was investigated in the Colombian Andes. Detailed examinations of inflorescence morphology, floral development and population phenology were conducted. Over a 1‐year period, flowering and fruiting phenology were recorded, the reproductive system was assessed through controlled pollination experiments, and spadix temperatures were monitored during anthesis. Floral scent samples were collected and characterized using headspace and Gas Chromatography coupled to Mass Spectrometry methods, while pollen morphology and its position on floral visitor’s body were described using scanning electron microscopy. Attractiveness assays with baited traps were employed to attract scent‐oriented floral visitors.

**Key results:**

The reproductive sequence of *S. weberbaueri* consists of six stages over 50 days. Anthesis, lasting three days, involves thermogenesis, spathe movements, floral chamber formation, and scent emission to attract and retain visitors. Temperature increases are synchronized with flowering, peaking at 5.4–7.2 °C above ambient during the male phase of anthesis. We identified 3-pentanol as a novel floral scent component in Araceae, which attracts *Cyclanthura* sp., the primary pollinators of *S. weberbaueri.* Controlled pollination experiments confirmed that *S. weberbaueri* cannot self-pollinate or undergo apomixis. We make the first report of brood-site pollination mutualism in *Stenospermation* and in the subfamily Monsteroideae.

**Conclusions:**

This is the first comprehensive account of the pollination biology of genera *Stenospermation*, indicating a mutual dependence between *S. weberbaueri* and *Cyclanthura* sp., with weevils serving as pollen vectors and laying eggs on deciduous spathes. This BSPM in aroids, involving ectophagous and detritivorous larvae, highlights the complex interplay of floral traits—centered on long—range olfactory signals combined with putative visual and thermal signals—to attract specialized pollinators.

**Supplementary Information:**

The online version contains supplementary material available at 10.1186/s40529-025-00468-w.

## Introduction

In the Araceae family, reproductive biology remains proportionally understudied due to the family’s high species diversity (> 3600 spp.), the complex morpho-anatomical features of its inflorescences and in some cases, the distribution in areas with difficult access, like forest canopy. Aroid inflorescences are defined by a spathe that envelops a spadix composed of minute unisexual or bisexual flowers (Boyce 1995; Mayo et al. 1997). In unisexually-flowered inflorescences, pistillate flowers are located at the base of the spadix, with staminate flowers at the top and sterile staminate flowers positioned between them to provide nutritional rewards for pollinators (Grayum 1990; Boyce 1995; Mayo et al. 1997). Enclosed within the spathe, the spadix functions as a site for mating while also offering food and shelter for floral visitors (Grayum 1990; Mayo et al. 1997; Roy and Raguso 1997; Mackay et al. 2014), and anthesis typically lasts 48–72 h (Gibernau et al. 2003; Pereira et al. 2014).

In contrast, hermaphrodite taxa exhibit flowers uniformly arranged on the spadix, and the spathe generally does not form a traditional pollinator chamber. In genera such as *Anthurium* and *Spathiphyllum*, anthesis may extend over several weeks (Franz 2007; Hentrich et al. 2010; Hartley and Gibernau 2019; Díaz et al. 2019; Rosero-Céspedes and Restrepo-Jaramillo 2019; Díaz-Jimenez et al. 2021). These species employ various strategies—including floral scent emission—to sustain regular pollinator presence during the extended anthesis period. Moreover, the chemical composition of aroid floral scents varies considerably among species, shaped by interactions with pollinators that reinforce scent—mediated reproductive isolation (Gibernau 2016) as well as by divergent phylogenetic forces and geographical distinctions (Friberg et al. 2019).

Research on pollination and chemical ecology in Neotropical Araceae has predominantly focused on a few key genera. Among those with unisexually-flowered inflorescences, *Philodendron* and *Xanthosoma* have received considerable attention. Species in these genera appear to be primarily pollinated by cyclocephaline scarab beetles (Melolonthidae, Dynastinae, Cyclocephalini) (Gibernau et al. 2000, 2021; Gibernau and Barabé 2002; García-Robledo et al. 2004; Maia et al. 2010, 2023; Pereira et al. 2014; Milet-Pinheiro et al. 2017). Their floral scents, crucial for attracting these nocturnal beetles, are often dominated by a limited number of major volatile organic compounds (VOCs). These include aliphatics (e.g., 2-hydroxy-5-methyl-3-hexanone), jasmone derivatives (e.g., (Z)-jasmone, isojasmol, dehydrojasmone), irregular terpenes (such as the apocarotenoid dihydro-β-ionone, and (E)-4,8-dimethylnona-1,3,7-triene and its derivatives), methoxylated benzenoids (e.g., 4-vinylanisole, methyl benzoate, methyl salicylate), and 2-alkyl-3-methoxypyrazines; many of these VOCs have demonstrated behavioral activity in attracting these beetles (Maia et al. 2010, 2023; Pereira et al. 2014; Milet-Pinheiro et al. 2017).

In contrast, investigations into genera with bisexually-flowered inflorescences have primarily addressed *Anthurium*, *Spathiphyllum*, and *Monstera*. *Anthurium* exhibits remarkable pollinator diversity, utilizing various bees (Euglossini, Meliponini, Tapinotaspidini), beetles (Curculionidae), and flies (Drosophilidae, Cecidomyiidae); Its floral scents are equally varied, ranging from unpleasant to sweet, and comprise a wide array of chemical classes including terpenoids, benzenoids, and lipid-derived compounds (Croat 1980; Montalvo and Ackerman 1986; Kraemer and Schmitt 1999; Chouteau et al. 2007; Franz 2007; Prieto and Cascante-Marin 2017; Etl et al. 2017; Díaz-Jiménez et al. 2021). This scent chemistry is pivotal, with variations potentially driving speciation through pollinator specificity. *Spathiphyllum* species are mainly pollinated by bees, particularly male euglossine bees collecting fragrant terpenoids and benzenoids (e.g., myrcene, ipsdienol, benzyl acetate, eugenol), and pollen-collecting bees like Apini and Meliponini. *Monstera* species are primarily pollinated by small nitidulid beetles, attracted by sweet or bittersweet odors, though detailed chemical analyses of their scents are still pending (Gibernau 2016). For other genera with bisexual flowers, such as *Stenospermation*, information on pollinators (infrequent fly visits reported) (Croat 1978; Gómez-Murillo and Cuartas-Hernandez, 2016) and floral scent chemistry remains very limited. This collective diversity underscores the complex evolutionary pathways of pollinator attraction within the Araceae, where floral scent chemistry often plays a decisive role in mediating these specialized interactions.

Among Araceae, the subfamily Monsteroideae remains one of the least taxonomically understood groups (Tam et al. 2004; Zuluaga et al. 2019; Cedeño-Fonseca et al. 2021; Croat et al. 2024a, b). Although members of this subfamily possess bisexually-flowered inflorescences, many genera exhibit short floral cycles and often form enclosed floral chambers. These chambers provide mating sites and shelter for pollinators, echoing strategies more typically associated with unisexually-flowered taxa (Chouteau et al. 2007; Díaz et al. 2019). Since Monsteroideae and Aroideae (unisexually flowered clade) subfamilies are not phylogenetically sister groups (Haigh, et al. 2023), this pollination strategy appears to have evolved at least twice (e.g. convergence) in the Araceae family . The Monsteroideae subfamily is composed of 12 genera among them the Neotropical genus *Stenospermation* is important with about 240 species (Castaño-Rubiano 2011; Zuluaga et al. 2019; Croat et al. 2024b). However, pollination ecology of *Stenospermation*, remains undocumented as just anecdotic reports (Gómez-Murillo and Cuartas-Hernandez, 2016) and suppositions about its probable pollinators had been made (Croat 1978).

The genus *Stenospermation* thrives across a wide altitudinal gradient—from sea level to 2200 m a.s.l.—with its highest diversity occurring between 400 and 1600 m a.s.l. in humid, rainy tropical forests. It exhibits considerable variation in spathe and spadix morphology, as well as in spathe longevity; traits that may significantly influence interactions with pollinators and thus shape pollination strategies, attraction mechanisms, and reproductive isolation. Despite this diversity and its potential ecological implications, the pollination ecology of *Stenospermation* remains largely undocumented. To begin addressing this critical knowledge gap and to understand how such morphological variability translates into functional pollination mechanisms, *Stenospermation weberbaueri* was selected for this foundational investigation. This species provides an opportunity to elucidate the fundamental processes governing pollinator interactions within this understudied genus.

The primary objective of this study is, therefore, to provide a comprehensive analysis of the key floral characteristics and reproductive biology of *Stenospermation weberbaueri* that mediate its pollination. To achieve this, our research specifically aims to: (i) Document the detailed floral morphology of *S. weberbaueri*, including pollen characteristics; (ii) Trace its complete floral development, from bud emergence through to fertilization and fruit production; and (iii) Determine how the release of floral volatiles and the occurrence of floral thermogenesis during anthesis contribute to the attraction of floral visitors and potential pollinators.

By systematically examining these aspects, this study will not only shed light on the specific ecological and reproductive dynamics underpinning pollination in *S. weberbaueri* but also aims to situate these findings within the broader context of pollination strategies across the genus *Stenospermation* and the Monsteroideae subfamily. This research is important as it will provide the first detailed insights into a representative species of a diverse yet poorly understood aroid genus, paving the way for future comparative studies.

## Materials and methods

### Study species

**Fig. 1:**
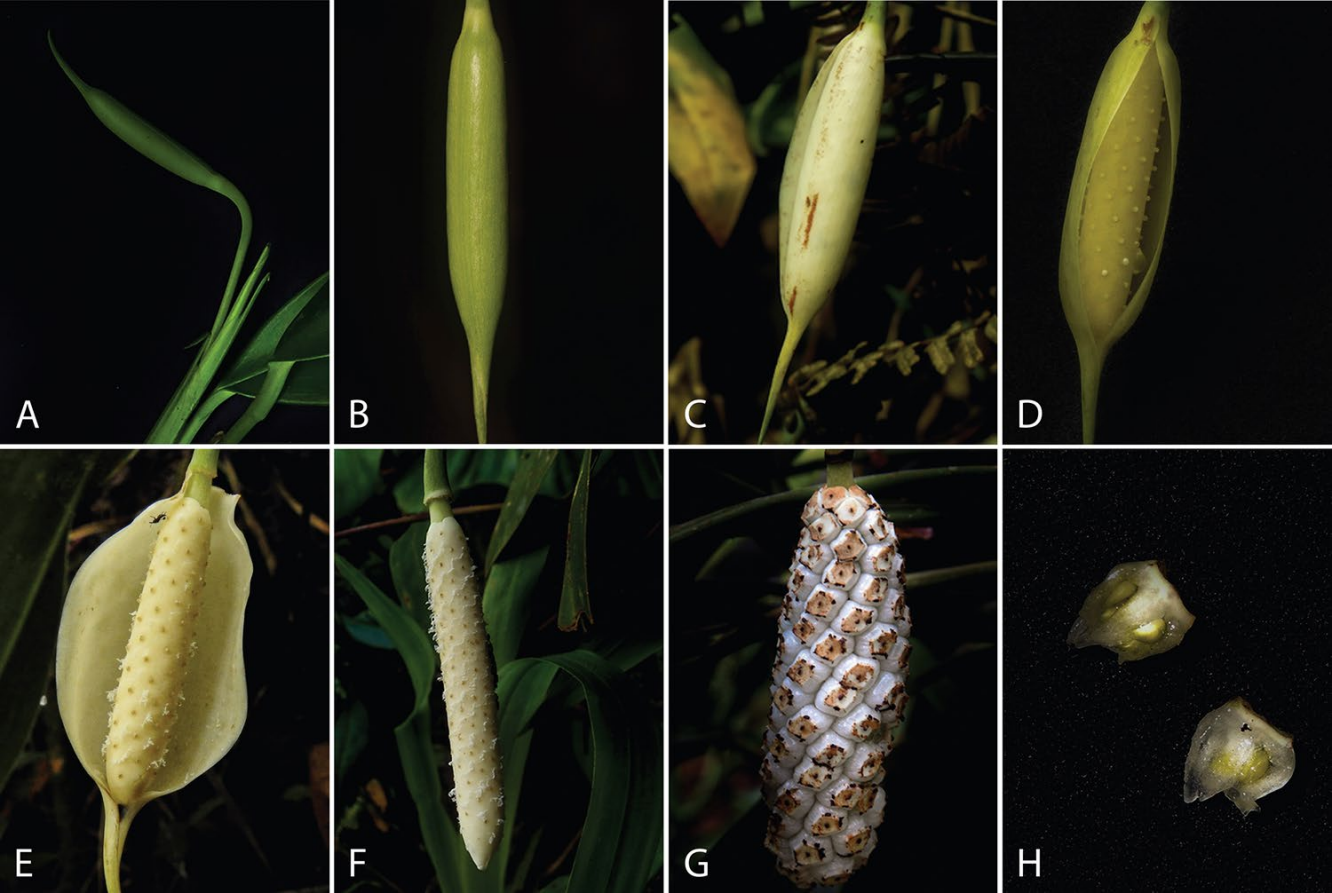
Floral morphology and inflorescence development in *Stenospermation weberbaueri*: **a** Flower bud **b** Immature inflorescence. **c** Female phase day one. **d** Female phase day two; note protruding stigmas; **e** Male phase, note pollen release; **f** Late male phase, note spathe dropped. **g** Mature infructescence; **h** Longitudinal section of fruit, showing two locus and seeds inside

*Stenospermation weberbaueri* is a medium-sized terrestrial to epiphytic herb (Zotz et al. 2021) distributed across tropical forests in Brazil, Colombia, Ecuador, French Guiana, Peru, and Venezuela (Castaño-Rubiano 2011). The inflorescences measure up to 45 mm in length, while the leaves may grow up to 50 cm. The lanceolate leaves feature acuminate apices, and the peduncles are sheathed by the preceding leaves; they are subtended and become cernuous at the apex. The spathes are white, as are the stipitate spadices, which are cylindrical and exhibit a conical apex (Fig. 1).

Botanical samples were collected to confirm taxonomic assignment. The exsiccates from these collections have been deposited in the CUVC herbarium at Universidad del Valle and the FAUC herbarium at Universidad de Caldas under the vouchers ANC 2303 and ANC 2377, respectively.

### Study site

Established in 2005 within a post-conflict zone, Selva de Florencia National Natural Park is one of Colombia’s best-preserved sub-Andean forest relicts (PNN 2019). Located between the eastern foothills of the Cordillera Central and the Magdalena River valley in eastern Caldas Department, the park covers 100.2 km^2^ across the municipalities of Samaná and Pensilvania. It includes the hydrographic basins of the La Miel and Samaná Sur rivers, both tributaries of the Magdalena River, and ranges in altitude from 850 to 2400 m.a.s.l. The predominant biome is Sub-Andean humid forest, with a smaller area of High-Andean humid forest (Herrera et al. 2018). The park plays a key role in regulating regional water resources and ranks among the most precipitation-rich areas in Colombia, with an average annual rainfall of 8000 mm (PNN 2019; Benavides-Ossa et al. 2022).

The investigated population of *S. weberbaueri* were observed in various habitats, including forested areas, secondary vegetation matrices, and grasslands near Bocatomas (n = 6 individuals) (from 5°31′18.5″N 75°03′36.2″W at 1756 m.a.s.l. to 5°31′20.5″N 75°03′39.5″W at 1762 m.a.s.l.) and La Estrella (n = 12 individuals) (from 5°31′2.40″N 75° 2′24.99″W at 1604 m.a.s.l. to 5°31′11.51″N 75° 2′31.27″W at 1672 m.a.s.l.).

### Inflorescence morphology and floral development

To assess floral morphology and developmental stages in *S. weberbaueri*, we systematically recorded data from January to December 2021, encompassing various floral phases. Using a digital caliper, we measured key parameters including spathe length and diameter, and spadix length. We also documented the spathe coloration, degree of opening, and persistence. Furthermore, we recorded the number of flowers per inflorescence (n = 10), the dimensions of individual flowers, and the number of seeds per fruit.

The complete floral development sequence, including anthesis, was meticulously documented by monitoring 117 inflorescences, 127 infructescences, 86 flowers, and 125 fruits originating from eighteen distinct individuals within our primary study population. To determine the precise timing and duration of anthesis for these individuals, plants were observed at multiple intervals throughout the day (e.g., every 1–2 h during expected peak activity and less frequently otherwise). This intensive observation schedule allowed us to record crucial events such as the onset and duration of floral thermogenesis, periods of volatile emissions, the presence and behavior of floral visitors, spathe opening and closing behavior, and stigmatic fluid secretion and pollen release. Additionally, by dissecting flowers and fruits at different developmental stages, we were able to document the timing of seed formation.

### Population phenology

Over the course of one calendar year (January to December 2021), we systematically monitored the reproductive phenology of the aforementioned eighteen individuals of *S. weberbaueri* at our study site. Observations were conducted at 20‐day intervals, during which we recorded both the presence or absence of inflorescences on each individual and their respective developmental stages (e.g., bud, female anthesis, male anthesis, post-anthesis, early fruit, mature fruit). This regular monitoring allowed us to determine the timing, duration, and synchrony of flowering and fruiting events within these populations.

To evaluate flowering synchrony, we employed the Activity Index method as described by D’Eça-Neves and Morellato (2004), following the application recommendations of Barbosa et al. (2020). Population-level synchrony was then classified based on the percentage of individuals flowering concurrently: asynchronous (< 20%), lowly synchronous (21–60%), or highly synchronous (> 60%), according to the criteria of Bencke and Morellato (2002). This quantitative approach provided valuable insights into the degree of temporal overlap in reproductive activity among individuals.

Seasonality was assessed through circular analysis using ORIANA v.4.02 software (Aleixo et al. 2014). In this analysis, each month was represented by a 30° angle, and we calculated an average vector (µ), its significance (via the Rayleigh test), and the average vector length (r). The average vector indicated the month with the highest probability of encountering flowering individuals, while the vector length (ranging from 0 for complete asynchrony to 1 for perfect synchrony) quantified the degree of flowering synchrony. For this analysis, developmental stages 1 to 5 were classified as flowering, whereas stage 6 was considered fruiting.

In addition, we incorporated precipitation data from the AQUARIUS Time-Series 20.2.207.0 weather station located within the study area. To examine the influence of rainfall on flowering patterns, we conducted a linear regression analysis using RStudio (version 4.2.2; R Core Team 2016).

### Spadix temperature

To investigate floral thermogenesis, we monitored the temperature of the spadix and the surrounding ambient air throughout the entire anthesis period (encompassing both female and male phases) of three different *S. weberbaueri* inflorescences. Temperature measurements were conducted using a Testo 175T3 two-channel data logger equipped with thermocouple sensors (accuracy: ± 0.1 °C). One sensor was carefully inserted approximately 5 mm deep into the spadix tissue near the base of the inflorescence to record internal temperature. The second sensor was positioned 50 cm away from the inflorescence to measure the ambient air temperature concurrently. Temperature readings from both sensors were automatically captured and logged at 5-min intervals, following the protocol detailed by Maia et al. (2010). In addition to the contact thermometry, we acquired thermal images of the inflorescences every 10 min during both female and male phases of anthesis using an infrared thermal camera (Vividia HT-19, Vividia Technologies, China; thermal resolution: 320 × 240 pixels) to visualize heat emission patterns.

### Sampling and chemical analyses of floral scents

We employed the dynamic headspace technique to extract volatile organic compounds (VOCs) emitted in situ by *S. weberbaueri* inflorescences during anthesis (Tholl et al. 2021). Individual inflorescences were enclosed in polyethylene terephthalate (PET) bags (Assarápido Cozinha 41 × 33 cm, WYDA, Brazil) that retained and concentrated the floral VOCs, while a small hole allowed continuous airflow to maintain equilibrium.

For VOC sampling, each bag was connected to a silanized glass cartridge containing 150 mg Tenax GR (composed of 2,6-diphenylene oxide and graphite sorbent particles, 80/100 mesh Supelco) (see Maia et al. 2021 for details). The cartridge was linked via silicone tubing to a vacuum pump (model G 12/01 EB, ASF Thomas, Inc., Germany) that extracted scented air at a constant flow rate of 250 mL/min for 20 minutes. After extraction, the cartridges were sealed with polyolefin film (Parafilm “M”, Heathrow Scientific) to protect the adsorbed compounds from environmental contaminants and excess moisture. The cartridges were then stored in cold boxes and transported to the laboratory within 24–72 hours, where they were maintained at − 18 °C in a sterile freezer until elution with solvent, which occurred within 30 days.

The VOCs were eluted from the cartridges using n-hexane (HPLC grade 95%, PanReac AppliChem) into 1.5 mL vials. The elution involved three consecutive washes over the Tenax GR—two washes with 0.05 mL of solvent followed by one wash with 0.1 mL—resulting in a total elution volume of 0.2 mL. For analysis, we used a Shimadzu GCMS-QP 2010 Plus gas chromatograph coupled with a mass spectrometer. Helium served as the carrier gas, and injections were performed in splitless mode at 230 °C. Separation was achieved on a Zebron- 5 column (30 m x 0.25 mm ID x 0.25 μm, Phenomenex^®^) with a 1 µL injection volume, 75 kPa pressure, and a flow rate of 1.34 mL/min. The temperature program started at 40 °C (held for 1 minute), increased at 6 °C/min to 250 °C (held for 1 minute), and concluded with a column cleaning phase at 300 °C (15 °C/min, held for 1 minute), for a total run time of 37 minutes. The ion source and interface temperatures were maintained at 280 °C and 300 °C, respectively, with electron impact ionization (EI) at 70 eV and a scan range of 40–500 m/z.

Data processing was performed using GCMS Solution version 4.3 (Shimadzu Scientific Instruments, Inc.), which enabled integration of the chromatogram peaks. The relative proportion of each VOC was calculated based on its peak area. VOC identification was accomplished by comparing experimental spectra with the NIST20 mass spectral library (similarity threshold >90%) and determining linear retention indices (RIs) using a homologous series of linear alkanes (C6–C40) according to the method of van Den Dool and Kratz (1963). The identity of the main constituent was confirmed by comparison with an authentic synthetic standard (3-pentanol, 98% chemical purity; Sigma-Aldrich, USA, reference P8025). All extractions and analyses were conducted at the Laboratorio de Cromatografía y Técnicas Afines, Universidad de Caldas.

### Floral visitors

Floral visitors were collected directly from inflorescences during both anthesis phases. Specimens were mounted, labeled, and photographed, and specialists identified them to the lowest taxonomic level by comparing the collected specimens with those housed at the Laboratorio de Colecciones Biológicas, Universidad CES. Voucher specimens were subsequently deposited at the Laboratorio de Colecciones Biológicas (Universidad de Caldas), Universidad CES, and the Colección del Museo de Entomología (Universidad del Valle).

To document visitor behavior, we monitored 107 inflorescences throughout anthesis, recording parameters such as arrival and departure times, duration of stay, overall activity, and the presence of pollen or stigmatic fluid. Observations were conducted during both day and night to determine precise visitation intervals and to assess interactions with spathe opening. During the female phase, small openings were made in the spathe with a scalpel to facilitate closer observation, and a subset of visitors were collected into vials containing 90% alcohol for taxonomic classification. High-resolution images of these visitors were obtained using a Nikon SMZ-1500 microscope coupled with a Nikon DS-U3 Digital Microscope Controller DS Ri1 at the Laboratorio de Imágenes, Facultad de Biología, Universidad del Valle. Additionally, select individuals were collected without alcohol preservation to directly assess the presence of pollen on their bodies.

Finally, to investigate the origins of minor feeding damage observed on the spathes, post- anthesis spathes were collected and placed in plastic bags lined with humid absorbent paper. These samples were maintained under environmental conditions for eight days prior to examination under a stereomicroscope.

### Pollen morphology and transport

Pollen grains from *S. weberbaueri* were collected during the male phase of anthesis for morphological characterization and to verify whether the pollen present on floral visitors matched. Pollen morphology was described following the methodologies of Grayum (1992) and Punt et al. (2007), and images were captured using a Quanta 250 scanning electron microscope (SEM) at the Instituto de Investigación en Estratigrafía (IIES), Universidad de Caldas.

### Bioassays with scent baits

To assess whether floral visitors are attracted to *S. weberbaueri* inflorescences based on olfactory cues, we conducted a semi-structured experiment using filter paper discs designed to mimic *S. weberbaueri* spathes (Boeco Germany, grade 3hw 56 g/m^2^). Each decoy spathe was fitted with a 2 mL amber silanized glass vial containing a cotton ball supported by a filter paper cone, onto which 0.2 mL of the corresponding solution was deposited. Six of these decoy spathes were treated with 0.2 mL of a synthetic standard corresponding to the main VOC identified in our analyses, while six others served as untreated controls.

Additionally, we collected six freshly abscised spathes and attached to each a 2 mL amber glass vial with cotton. Three of these spathes were treated with 0.2 mL of the main VOC from the floral scent of *S. weberbaueri*, and three were left untreated as controls. The volume of 0.2 mL was sufficient to make the aroma perceptible to the human nose, and were refilled when the aroma was no longer perceptible. All experimental treatments were positioned near *S. weberbaueri* individuals during the scent emission period of anthesis, with each trap spaced 1–1.5 meters apart, for a period of one hour.

### Reproductive system

To accurately designate the reproductive system of *S. weberbaueri*, we adhered to the nomenclature outlined by Cardoso et al. (2018) and performed bagging experiments following the methods of Till‐Bottraud (1993) and Chouteau et al. (2006). Specifically, we enclosed ten unopened inflorescences in fine veil fabric bags to prevent access by floral visitors, while a control group of 266 inflorescences was left unbagged. Over the following 3 months, we monitored fruit set in both groups and compared the development of the bagged inflorescences with that of the unbagged controls to accurately assess the reproductive system.

### Used of large language models (LLMs), such as ChatGPT

As none of the authors are native English speakers, we used ChatGPT to check the coherence, grammar, and overall language clarity.

## Results

### Inflorescence morphology and floral development

We found that the inflorescences of *Stenopermation weberbaueri* contained between 120 and 152 flowers, forming about the same number of fruits. Each fruit contained one to four seeds. As well as, we defined six stages of inflorescence development (Fig. 1, Table 1): i. Flower bud, enclosed within the blade sheath; ii. Immature inflorescence, reaching final size but green and immature; iii. Pre-anthesis, with the spathe lightening in color; iv. Anthesis, where the spathe fully opens exposing flowers; v. Post-anthesis, with flowers unreceptive and pollen already dispersed; vi. Infructescence, where fertilized flowers develop into fruits. The transition from stage one to five takes 50 days (flower bud to post-anthesis), followed by a 20-day period for fruit ripening initiation.

**Table 1:** Floral morphology of *Stenospermation weberbaueri* from specimens growing in Selva de Florencia National Natural Park, Caldas department, Colombia

Structure	Measurements (mm)	
	Length	Width
Inflorescence in bud (2)	61.55 ± 9.26	6.9 ± 2.96
Immature inflorescence (6)	63.45 ± 11.41	9.98 ± 6.43
Inflorescence in anthesis (2)		
Spathe	64.5 ± 3.53	32.5 ± 0.70
Spadix	47.5 ± 2.12	8.0 ± 0.0
Inflorescence in post-anthesis (8)	45.98 ± 5.47	8.51 ± 0.73
Developing infructescence (16)	41.5 ± 52.4	8.68 ± 1.48
Flowers (85)		
Ovary	2.18 ± 0.47	2.01 ± 0.39
Stylar zone	3.18 ± 0.56	2.52 ± 0.53
Stigma	0.62 ± 0.36	0.43 ± 0.26
Locule	1.24 ± 0.35	1.54 ± 0.42
Theca	0.67 ± 0.23	0.45 ± 0.15
Filament	1.30 ± 0.55	0.97 ± 1.985
Fruits (127)	3.93 ± 2.54	3.24 ± 0.77
Seeds (229)	2.13 ± 0.22	1.21 ± 0.23

Measurements (in mm) are presented as mean ± standard deviation, and sample size for each character are shown in parentheses

During stage iv (anthesis), lasting three days, the first two days encompass the female phase. Initially, the spathe undergoes a change, becoming detached from the spadix and turning white internally, while externally shifting from green-yellowish to whitish with a white margin. The spathe partially opens, revealing the mostly concealed spadix with elevated stigmas. This initial opening lasts four hours, starting at 14:00 and ending at 18:00, when, the spathe closes and remains closed for the next 20 h. The next day, at 14:00, the spathe opens again, revealing the ivory-colored spadix, with flowers displaying raised stigmas coated with stigmatic fluid. This phase concludes around 18:00, when the spathe closes again for the last time. On the third day at 14:00, the spathe opens again, fully revealing the spadix. The flowers now have unreceptive stigmas, and pollen is released in strands resembling a pollen kit extrusion (Fig. 1) **[Supplementary Information video 1].** The stamens are not visible as they are shorter than the flowers. Throughout this period, the spathe gradually opens completely and detaches by around 18:00.

### Population phenology

**Fig. 2:**
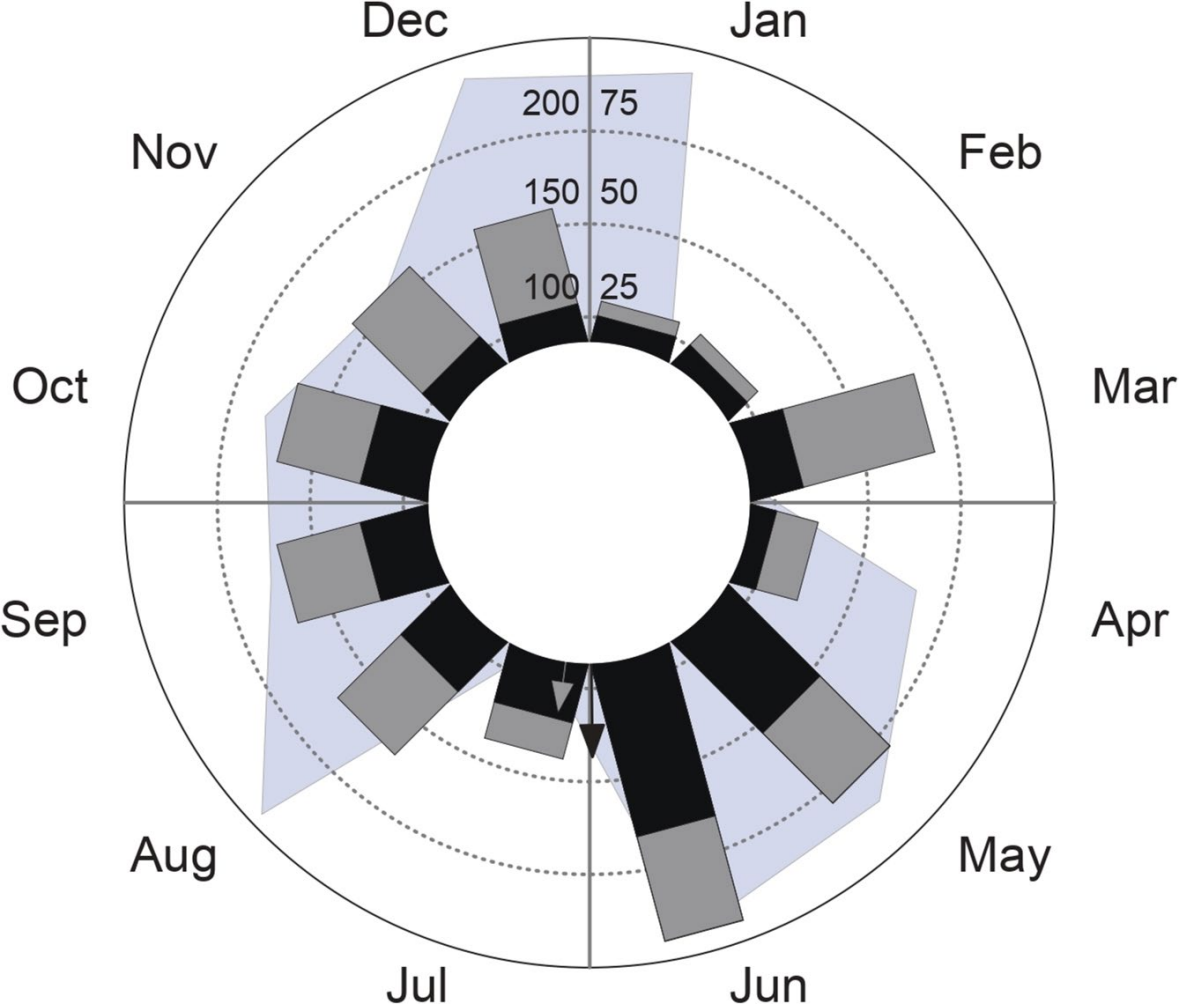
Annual flowering (gray) and fruiting (black) phenology in a population of *Stenospermation weberbaueri* in sub-Andean Colombia (n = 18 individuals). On the vertical (y) axis, values to the right correspond to the number of reproductive structures (inflorescences, gray; infructescences, black), represented as columns. Values to the left refer to accumulated rainfall (in mm), shown in the graph as the light blue polygon. Arrows correspond to Rayleigh test vectors (*r*), indicating the interval with the highest probability of encountering flowering or fruiting individuals

During the sampling period, a total of 271 reproductive structures were observed (Flowers and fruits). The investigated population of *S. weberbaueri* has a continuous, year-round flowering and fruiting behavior (length of mean vector *r* = 0.186 and 0.320, respectively). The peak reproductive activity for both flowering and fruiting was discreetly more pronounced in May–June, while January–February exhibited lower rates. Neither flowering nor fruiting correlate with monthly precipitation levels (Fig. 2).

### Spadix temperature

In *S. weberbaueri*, thermogenesis begins in the afternoon (around 14:00) on the first day of the female phase of anthesis, when the spathe is nearly closed. At this stage, the temperature of the inflorescence rises by 1–2 °C above the ambient air temperature. This temperature pattern continues into the following day as the spathe begins to open.

However, during the afternoon of the third day, corresponding to the male phase of anthesis, the spadix exhibits temperature increases ranging from 2.7 to 5.4 °C above the ambient air temperature, reaching up to 24.7 °C. These thermogenic peaks coincide with the major emission of floral scent (detected by sniffing and sampling) and are synchronized with the reported period of anthesis.

**Fig. 3:**
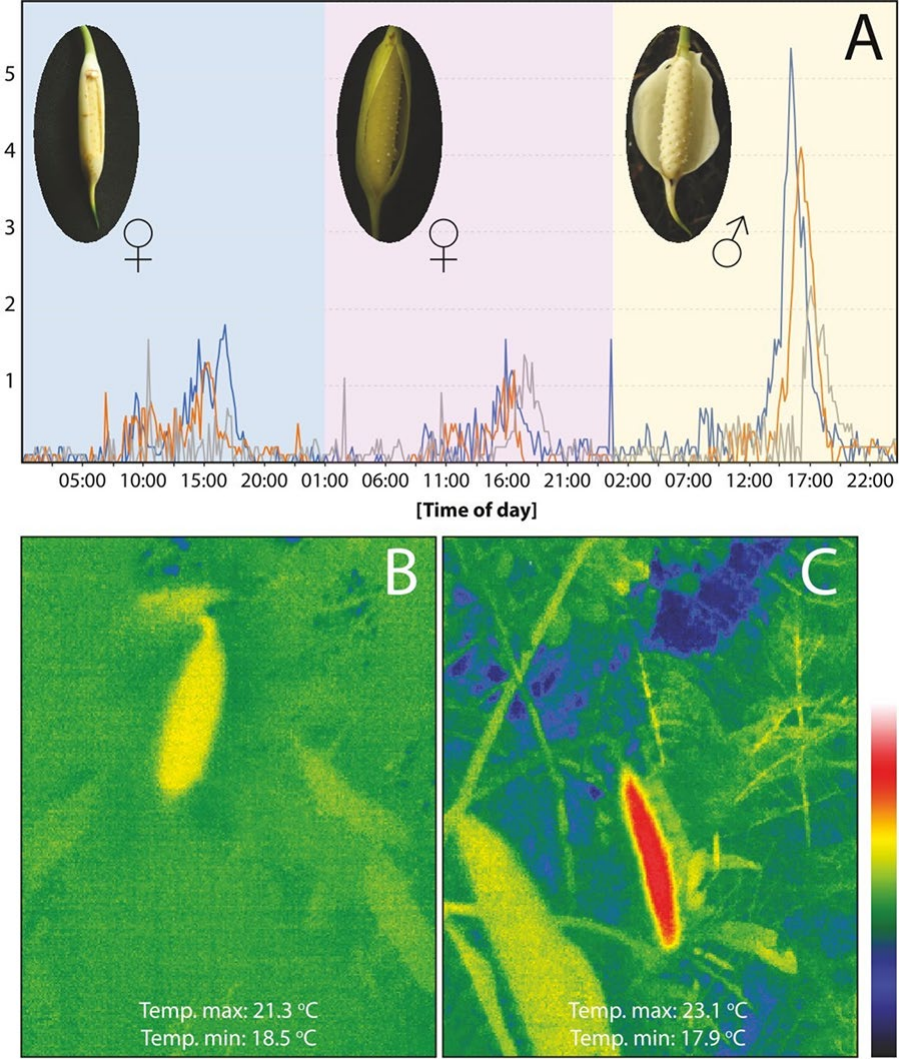
Floral thermogenesis in *Stenospermation weberbaueri*: **a** Graph showing temperature variations over the three days of anthesis. The inflorescences are depicted for each day of anthesis; the red, blue, and brown lines represent the thermal variations of three different inflorescences, **b** Thermal images during the second day of the female anthesis phase, and **c** Thermal images during the male anthesis phase

These findings are supported by thermal imaging camera data, which recorded temperature increases of up to 7.2 °C above the surrounding air temperature during both phases of anthesis (Fig. 3).

### Floral visitors

**Fig. 4:**
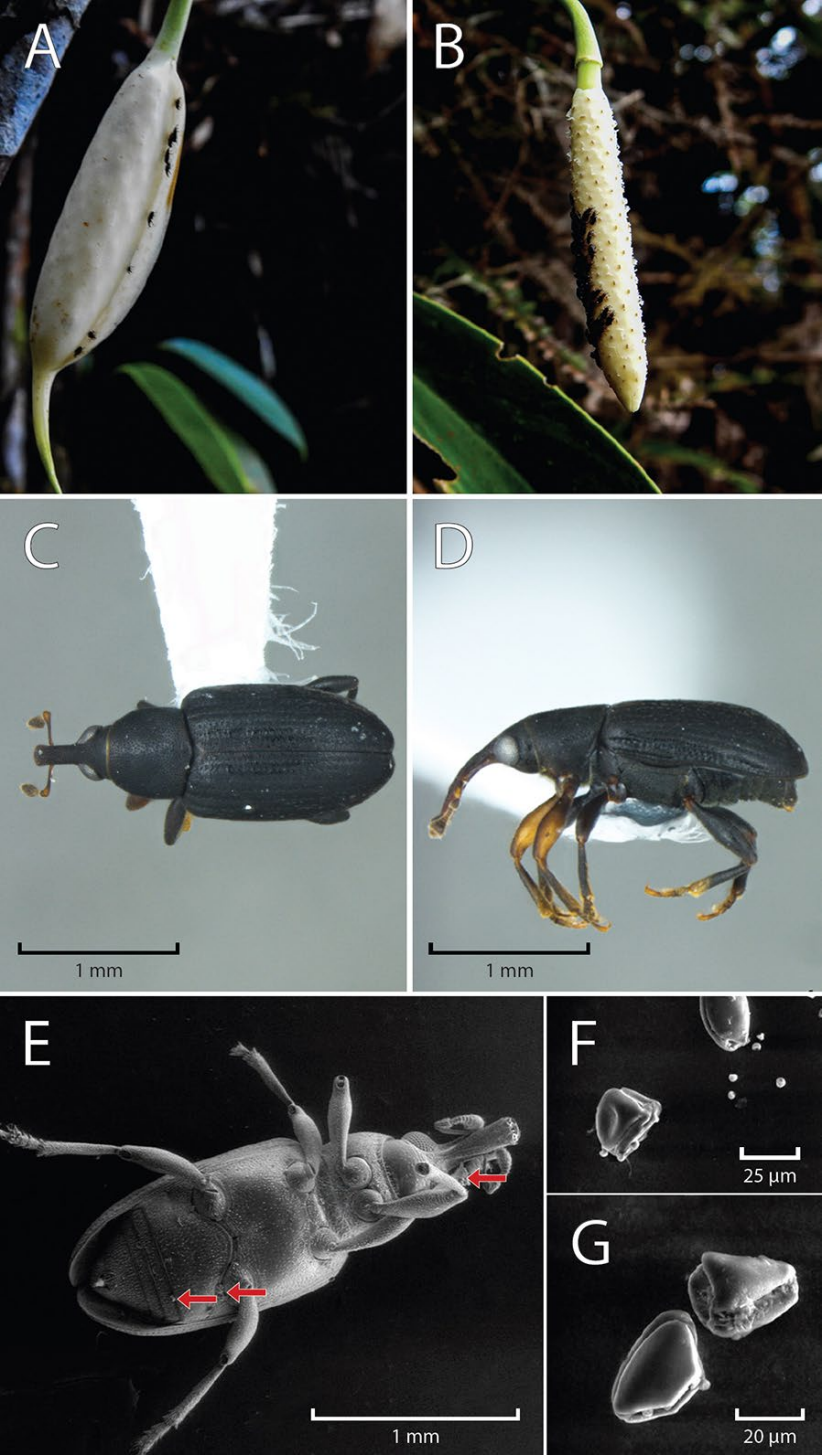
Floral visitors and pollen morphology in *Stenospermation weberbaueri*: Individuals of the flower weevil *Cyclanthura* sp. (Curculionidae, Derelomini) on inflorescences during the **a** female and **b** male phases of anthesis. **c** Dorsal and **d** lateral view of *Cyclanthura* sp. **e** Pollen attached to the visitor’s body; red arrows show pollen masses. **f**-**g** Pollen morphology

A single morphospecies of *Cyclanthura* (Curculionidae, Derelomini) was identified as the sole floral visitor associated with *S. weberbaueri* throughout our study, with an average of 50 individuals recorded per inflorescence (Fig. 4a–d). Our findings constitute the first report of Curculionidae as pollinators in *Stenospermation* and within the subfamily Monsteroideae. These tiny weevils arrived at the onset of anthesis during the female phase (around 14:00) and remained within the spathe until the male phase, departing shortly after the spathe dropped **[Supplementary Information video 1 and Supplementary Information video 2]**.

During both phases of anthesis, *Cyclanthura* sp. weevils moved around the spadix in search of stigmatic fluid, bringing them into contact with the reproductive structures; copulation was also observed **[Supplementary Information video 3]**. The weevils inserted their rostra into narrow spaces between the flowers, causing pollen grains to adhere to their bodies (Fig. 4).

**Fig. 5:**
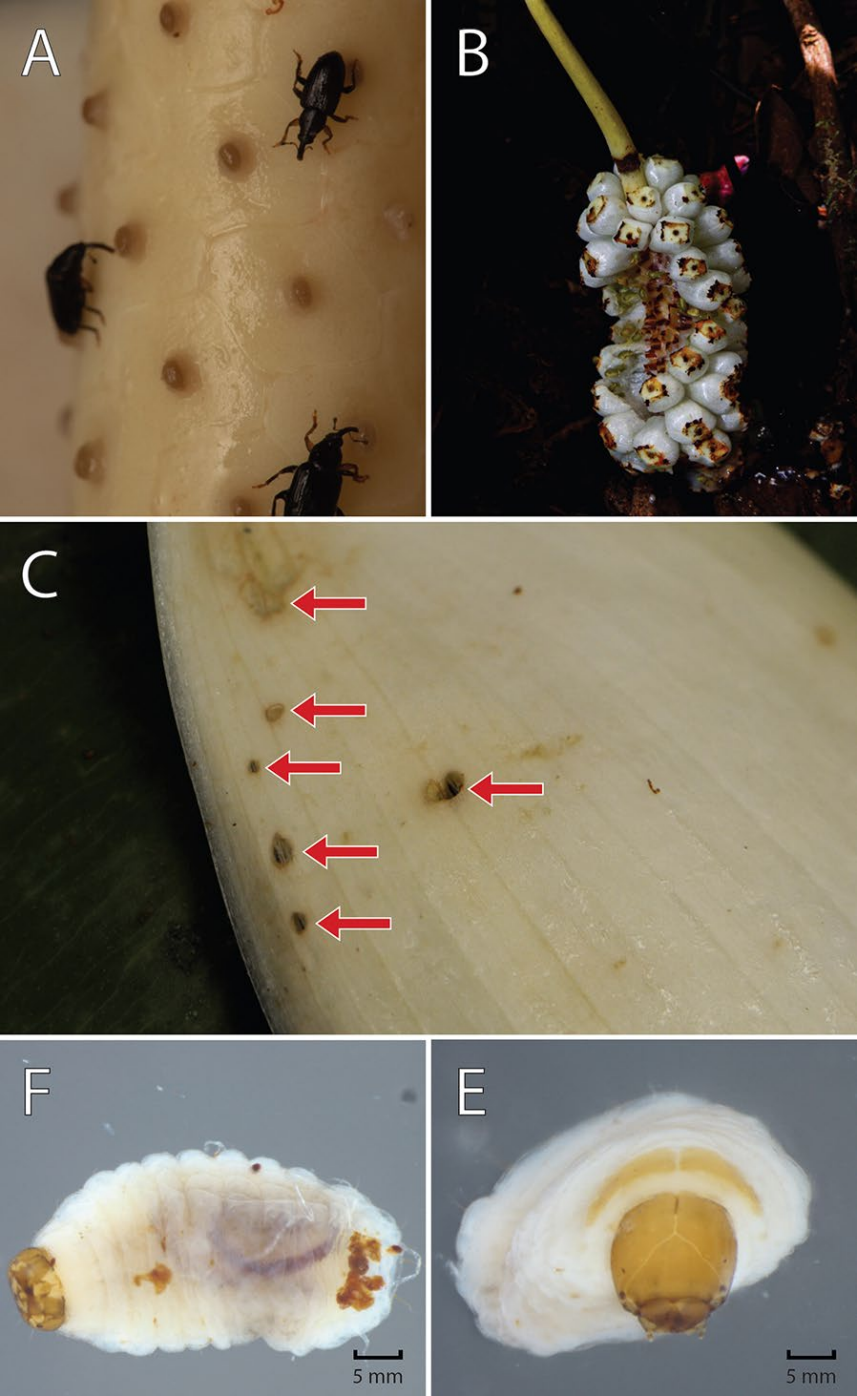
Ecological interactions of *Stenospermation weberbaueri*: **a** Individuals of the flower weevil *Cyclanthura* sp. (Curculionidae, Derelomini) on flowers. **b** Mature infructescence partially consumed by fruit dispersers. **c** Damage on the inner surface of a recently fallen spathe (red arrows). **d** Ventral and **e** frontal view of a larva of *Cyclanthura* sp. recovered inside the spathe

Additionally, we observed distinct ecological interactions facilitated by *S. weberbaueri* inflorescences, which offer multiple floral resources to these specialized floral visitors (F igure 5). These resources include stigmatic fluids during female anthesis, shelter, and warmth throughout anthesis, thanks to the protective floral spathe forming a ‘pollination chamber’ and floral thermogenesis. The inflorescences also serve as copulation sites, as evidenced by mating behavior, and as oviposition substrates, with larvae found in various developmental stages within decaying spathes, though never on the spadix (Fig. 5c–e).

### Pollen morphology

*Stenospermation weberbaueri* pollen grains are monads, peroblate to semi-triangular convex, meridionosulcate, with exine that is psilate to punctate. Their equatorial view length ranges from 30 to 37 µm (Fig. 4f–g). Notably, pollen grains were observed clinging to the legs and rostra of flower-visiting weevils (Fig. 4e).

### Floral scent chemistry and bioassays with scent baits

**Fig. 6:**
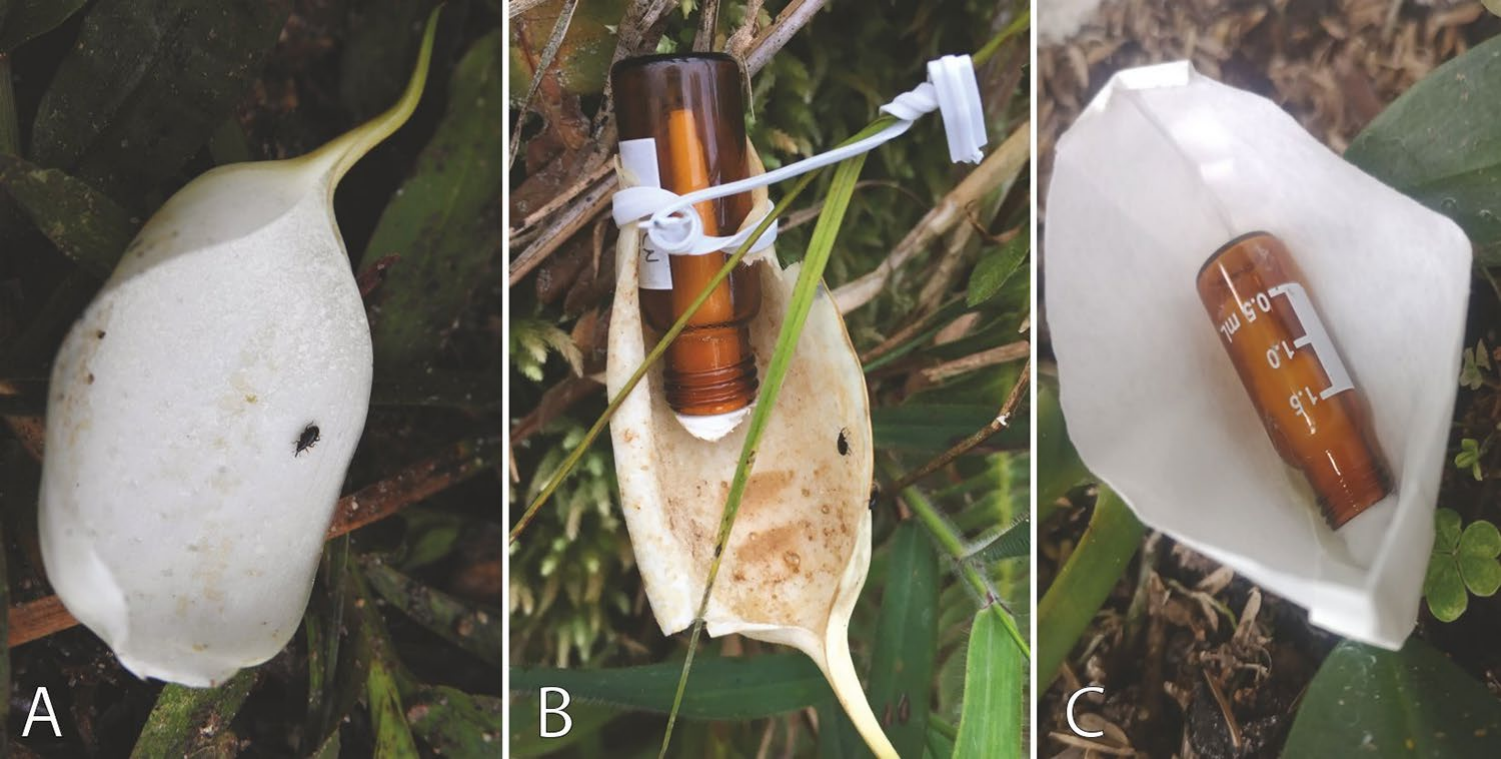
Field attractiveness assays: **a** Natural *Stenospermation weberbaueri* spathe. Notice the attracted Cyclanthura sp. weevils (Curculionidae, Derelomini). **b** Natural spathe with an attached vial baited with 3-pentanol. Notice the attracted Cyclanthura sp. weevils (Curculionidae, Derelomini). **c** Baited filter paper decoy spathe.

The floral scent profile of *S. weberbaueri* during both phases of anthesis is dominated by a single ion peak with a Kovats RI of 690, identified as the aliphatic alcohol 3-pentanol. *Cyclanthura* sp. weevils were observed flying around (and occasionally landing on, one individual) all filter paper decoy spathes baited with 3-pentanol. They were similarly attracted to and landed on natural spathes, whether baited (two individuals) or unbaited (one individual). In contrast, unbaited filter paper decoy spathes did not attract the weevils (Fig. 6; Table 2).

**Table 2:** Attractiveness assays for *Cyclanthura* sp. flower weevils (Curculionidae, Derelomini), pollinators of *Stenospermation weberbaueri* (Araceae), using 3-pentanol

Type of trap	N^o^ weevils landed	Weevils flying closely
Scent-baited filter paper decoy	1	Yes
Natural spathe	1	Yes
Scent-baited natural spathe	2	Yes
Unbaited filter paper decoy	0	No

Decoys were assembled from folded filter paper discs to resemble open spathes

### Reproductive system

**Fig. 7:**
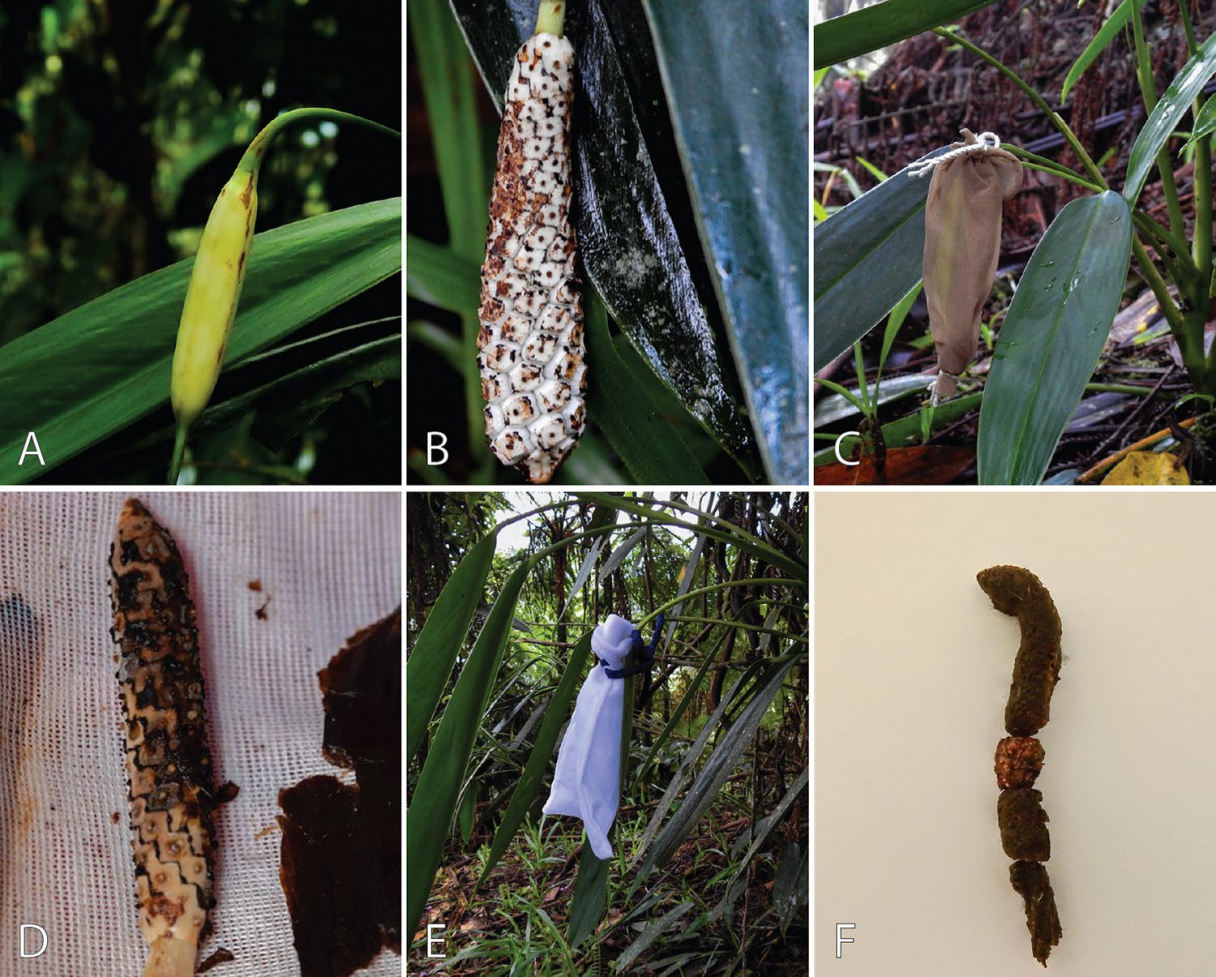
Pollination tests on *Stenospermation weberbaueri* inflorescences: **a** Unbagged control inflorescence, **b** Fruit ripening in unbagged inflorescence, **c**, **d** Bagged inflorescences, **e**, **f** Inflorescences in post-anthesis decomposition

None of the bagged inflorescences (*n* = 10) produced infructescences. This indicates avoidance of spontaneous self-pollination through perfect protogyny. In contrast, all marked and control inflorescences produced infructescences, suggesting that cross-pollination predominates under natural conditions and is highly efficient (Fig. 7).

## Discussion

One of the main findings of this study is that *S. weberbaueri* relies on specialized insect pollen vectors, as confirmed by controlled pollination experiments. This dependence is reflected in specific morphological and physiological traits, including thermogenesis during anthesis and the formation of a pollination chamber (Maia and Schlindwein 2006; Gibernau and Barabé 2002; Maia et al. 2010, 2012; Pereira et al. 2014; Gibernau et al. 2021). These adaptations parallel those reported in two *Monstera*—another Monsteroideae genus featuring short floral cycles and enclosed inflorescences pollinated by Nitidulidae beetles (Chouteau et al. 2007; Prieto and Cascante-Marín 2017). But in *S. weberbaueri*, these convergent floral traits have evolved in relation with another pollinator group, the Derelomine flower weevils. Both, floral volatiles and thermogenesis plays central roles in its pollination system, mirroring the pollination chambers of *Monstera* (Chouteau et al. 2007, 2009; Prieto and Cascante-Marín 2017) that provide a protected environment for insect visitors (Roy and Raguso 1997; Gibernau and Barabé 2000, 2002; Mackay et al. 2014).

Our observations confirm that the spathe of *S. weberbaueri* acts as a functional pollination chamber, opening and closing in synchrony with anthesis to retain pollinators and enhance reproductive success. The overall duration of anthesis is relatively brief—similar to the short cycles seen in unisexually-flowered aroids (Gibernau et al. 2003; Pereira et al. 2014)—and contrasts with the protracted anthesis (lasting days or weeks) found in bisexually—-flowered genera like *Anthurium* and *Spathiphyllum* (Franz 2007; Hentrich et al. 2010; Díaz et al. 2019; Rosero-Céspedes and Restrepo-Jaramillo 2019; Díaz-Jimenez et al. 2021). Other short—-anthesis genera with unisexual flowers include *Montrichardia* and *Philodendron*, where a similar open-close spathe mechanism spans 48–72 h (Gibernau et al. 2003; Pereira et al. 2014), while *Furtadoa* and *Schismatoglottis* complete anthesis in roughly one day (Mori and Okada 2001; Hoe et al. 2016). Although *Homalomena* extends its anthesis to 53–62 h, its female phase remains confined to the first day (Hoe et al. 2016).

Because *S. weberbaueri* lacks self-pollination and apomixis, its reproduction hinges on outcrossing assisted by pollen vectors. This stands in contrast to other bisexually-flowered Monsteroideae in which self-pollination or apomixis may occur (Chouteau et al. 2007; Hentrich et al. 2010; Prieto and Cascante-Marín 2017).

Regarding floral thermogenesis, spadix temperature peaks coincide with the spathe’s opening during both female and male phases of anthesis. During the female phase, temperatures track ambient fluctuations, while in the male phase, temperature surges exceed 5 °C above ambient, mirroring the thermogenic patterns reported in *Monstera* (Chouteau et al. 2007, 2009; Prieto and Cascante-Marín 2017). This thermal elevation corresponds with increased floral scent emission, stigmatic receptivity, and pollen release, in line with other documented thermogenic aroids (Gibernau et al. 2000, 2003; Gibernau and Barabé 2002; Maia and Schlindwein 2006; Chouteau et al. 2007, 2009; Bröderbauer et al. 2014; Prieto and Cascante-Marín 2017).

The exclusive association of *Cyclanthura* sp. weevils with *S. weberbaueri* inflorescences, along with their high abundance, continuous presence throughout anthesis, consistent interaction with reproductive structures, and visible pollen loads, confirms their effectiveness as pollinators. While many weevil species (Curculionidae) have historically been labeled as agricultural pests (Anderson 1995; Fernández and Fernández 2010; Piñero and Ruíz 2012; Haran et al. 2023), others serve critical ecological functions as pollinators in tropical plant families such as Araceae, Arecaceae, and Cyclanthaceae (Eriksson 1994; Chouteau et al. 2007; Franz 2007; Núñez-Avellaneda and Rojas 2008; Gibernau 2011; Núñez-Avellaneda et al. 2015; Guerrero-Olaya and Núñez-Avellaneda 2017; Cortes and Gómez 2018; Cortés et al. 2018). For example, some *Anthurium* species appear to rely solely on derelomine flower weevils for pollination (Franz 2007), and larval development on or within spathes is well documented in multiple weevil species (Haran et al. 2023).

The dominance of 3-pentanol in the floral scent of *S. weberbaueri* is consistent with patterns seen in other beetle-pollinated aroids like *Philodendron squamiferum* (Maia et al. 2019) and *Xanthosoma riparium* (Milet-Pinheiro et al. 2017), as well as certain species with specialized pollination systems (e.g., *Syngonium hastiferum*, pollinated by mirid bugs; Etl et al. 2017). An extensive survey suggests that 3-pentanol has not been reported in any other Araceae species (Knudsen et al. 2006; El-Sayed 2024). Notably, 3-pentanol functions as a sex pheromone in the ambrosia beetle *Megaplatypus mutatus* (Funes et al. 2011) and can also trigger systemic resistance in plants (Song et al. 2015). Our bioassays indicate that *Cyclanthura* sp. Weevils respond preferentially to 3-pentanol support its role in specialized pollinator attraction.

Although *Cyclanthura* sp. weevils were attracted by 3-pentanol in field tests, their tendency not to land on decoys suggests that additional cues—such as the high reflectivity of the spathe or the inflorescence’s thermal properties—may also play a role in pollinator orientation. This aligns with other cases where visual or thermal cues synergize with olfactory signals, such as in the deceptive pollination system of *Helicodiceros muscivorus* (dead-horse arum; Angioy et al. 2004) or in the mutualistic *cyclocephaline* beetle pollination of *P*. aff. *selloum* (Gottsberger et al. 2013).

During field observations, we consistently observed adult *Cyclanthura* sp. weevils interacting with *S. weberbaueri* inflorescences, and larvae were frequently observed on fallen and decaying spathes. These larvae appeared to feed externally on decaying floral tissues, without affecting ovary or seed development. These patterns suggest that *S. weberbaueri* participates in a brood-site pollination mutualism (BSPM), where spathes serve both as sites for larval development and floral structures for pollen transfer. In this mutualism, adult weevils act as pollen vectors, while their larvae feed on senescent tissues, a behavior consistent with ectophagous and detritivorous feeding habits. This arrangement aligns closely with the BSPM systems described by Sakai (2002), in which pollinators utilize decaying floral tissues for brood development without damaging reproductive organs, thus preserving plant health, thereby maintaining the mutualistic balance which is critical to the stability of BSPM and differentiates them from antagonistic or parasitic interactions. Our observations of weevil larvae in fallen, decaying spathes suggest ectophagous, detritivorous feeding habits that do not impair the plant’s reproductive success. Our study expands the known diversity of BSPM, as this is, to our knowledge, the first documentation of such a mutualism in the genus *Stenospermation* and in the subfamily Monsteroideae, and raises important questions about its prevalence in the genus *Stenospermation.* Additional comparative studies across the genus would be needed to determine whether this interaction represents a broader evolutionary trend within *Stenospermation* or a lineage-specific adaptation. Moreover, the evolutionary implications of BSPM are significant. This system involves tight coordination between floral phenology, morphology, and the life cycle of pollinating insects, suggesting a long history of co-evolution. The apparent specificity between *S*. *weberbaueri* and *Cyclanthura* sp. may point toward a co-adapted relationship, where floral traits such as scent profile, spathe architecture and behavior, and timing of anthesis are finely tuned to pollinator behavior and larval development requirements.

Overall, our study provides the first comprehensive account of the pollination biology of genera *Stenospermation* , building upon earlier observations and hypotheses (Croat 1978; Gómez-Murillo and Cuartas-Hernandez 2016). Likewise, the species exhibits high fruit set, indicative of successful reproduction in the study region, and underscores the importance of specialized weevil pollinators in maintaining the reproductive ecology of Monsteroideae aroids and highlight the evolutionary and ecological significance of BSPM as a mutualistic strategy.

## Fundings

We thank the Universidad de Caldas and their project “Análisis de compuestos orgánicos volátiles en un ensamblaje de aráceas en el Parque Nacional Natural Selva de Florencia (Samaná, Caldas) mediante DHS-GC/MS” (code 0277420) for their funding. This study was conducted under the “Permiso Marco de Recolección, Resolución 1070 del 28 de agosto de 2015 y Resolución 01004 del 7 de junio de 2019, hoja 8,” and the “Permiso de Parques Nacionales Naturales, Resolución No. 023 del 10 de marzo de 2021.”

## Ethics approval and consent to participate

All plants collections were made in accordance with ethics and academic’s regulations in Colombia legislation.

## Supplementary Information

Additional file 1 (Supplementary Information-Video 1: Dropping spathe of S. weberbaueri during male phase of anthesis. Notice the Cyclanthura sp. weevils avoiding direct light exposure.)

Additional file 2 (Supplementary Information-Video 2: Cyclanthura sp. weevils arriving at inflorescences of S. weberbaueri during the female phase of anthesis.)

Additional file 3 (Supplementary Information-Video 3: Cyclanthura sp. weevils in inflorescences of S. weberbaueri eating and entering in contact with stigmas and pollen.)

## Data Availability

The data used in this study were originally collected by the authors and are available from the corresponding author upon reasonable request.
